# The Technological Development of Minimally Invasive Spine Surgery

**DOI:** 10.1155/2014/293582

**Published:** 2014-05-21

**Authors:** Laura A. Snyder, John O'Toole, Kurt M. Eichholz, Mick J. Perez-Cruet, Richard Fessler

**Affiliations:** ^1^Barrow Neurological Institute, Phoenix, AZ 85013, USA; ^2^Rush University Medical Center, Chicago, IL 60612, USA; ^3^St. Louis Minimally Invasive Spine Center, St. Louis, MO 63141, USA; ^4^Michigan Head and Spine Institute, Southfield, MI 48034, USA

## Abstract

Minimally invasive spine surgery has its roots in the mid-twentieth century with a few surgeons and a few techniques, but it has now developed into a large field of progressive spinal surgery. A wide range of techniques are now called “minimally invasive,” and case reports are submitted constantly with new “minimally invasive” approaches to spinal pathology. As minimally invasive spine surgery has become more mainstream over the past ten years, in this paper we discuss its history and development.

## 1. Introduction

Although humans have attempted to treat spinal pathology since the times of Hippocrates and Paul of Aegina, minimally invasive spine surgery has only been recently developed in the past 50 years. However, in that time, remarkable innovation has occurred in terms of the indications for its use and the procedures performed. The goal of minimally invasive surgery, to reduce iatrogenic tissue trauma and thus reduce resultant postoperative pain and disability for patients, is one appealing to patients and surgeons alike. We will describe in this paper some of the major developments in minimally invasive spine surgery. However, we must emphasize that there is no specific timeline for minimally invasive spine surgery as many of these developments occurred concurrently and interdependently.

## 2. Improvements in Visualization Improving Technique

### 2.1. Spinal Endoscopy

Some of the earliest advancements in attempting to create a more minimally invasive spine procedure stemmed from improving visualization. In 1931, Burman introduced the concept of myeloscopy for direct spinal cord visualization [1]. In 1938, Pool expanded on Burman's work of myeloscopic inspection of the cauda equina and in 1942 introduced the concept of intrathecal endoscopy. He reported the results of more than 400 myeloscopic procedures [2, 3]. Myeloscopy fell out of favor for a time because of the morbidity associated with insertion of a large-bore scope into the dural cavity. The state of spinal endoscopy remained essentially the same until Ooi et al. [4] used an endoscope to examine the intrathecal space before surgery. Using improved technology, Ooi et al. [5] were able to describe pathological features in greater detail, including chronic arachnoiditis and nerve root excursion during claudication associated with lumbar spinal stenosis.

### 2.2. Thoracoscopic Spine Surgery

Jacobeus, a professor of internal medicine in Stockholm, Sweden, is credited with performing the first thoracoscopic procedure in 1910 [6]. As an internist, his major aim was to observe the pleural space in the diagnosis and treatment of pulmonary tuberculosis. After his initial diagnostic procedure, Jacobeus described the technique of lysis of tuberculous pleural adhesions, which was performed with a cystoscope and a heated platinum loop [7]. In 1990, with the introduction of video imaging to standard endoscopy, the modern era of thoracoscopy began. Mack et al. [8] in the United States and Rosenthal et al. [9] in Europe first reported the technique of video-assisted thoracoscopic surgery (VATS) in 1993 and 1994. Thoracoscopic spine procedures were initially implemented for disc herniations, sympathectomies, pathologies of the vertebral body, abscess drainage, and tumor biopsies. In the ensuing years, it has been implemented for scoliosis correction, anterior interbody fusion, osteotomies and bone grafting, corpectomies, and vertebral instrumentation in the treatment of tumors and fractures.

### 2.3. Percutaneous Arthroscopic Discectomy

Ottolenghi [10] in Argentina in 1955 and Craig [11] in 1956 described posterolateral biopsy of the spine. In 1975, Hijikata et al. [12] demonstrated a percutaneous nucleotomy by utilizing intradiscal arthroscopic techniques for disc removal in the treatment of posterior or posterolateral lumbar disc herniations under local anesthesia. After discography using Evans blue dye, specifically designed instruments were placed in a 5 mm cannula and inserted against the lateral annulus. A circular incision was made in the annulus, and the blue-stained nucleus pulposus was removed with pituitary forceps. Refinements to the technique involved the use of an automated system.

In 1983, Kambin and Gellman [13] performed a discectomy by inserting a Craig cannula and a small forceps into the disk space after an open laminectomy to evacuate the nucleus pulposus and observed the effects on the surrounding anatomic features. In 1985, Onik et al. [14] reported the development of a 2 mm blunt-tipped suction-cutting probe for automated percutaneous discectomy at L4-L5 or higher levels. Their reported outcomes indicate an overall success rate of 75%, with a complication rate of 1%.

Subsequent developments led to the design of a 2.7 mm glass arthroscope combined with a videodiscoscope with a single working portal [15]. This development enabled observation of periannular structures, including the foramen and the spinal nerve. Arthroscopic disc surgery allows removal of herniated discs via a posterolateral approach. This is accomplished with biportal access via triangulation into the intravertebral disc with inline irrigation and suction [15].

Numerous studies on the efficacy of arthroscopic disc surgery have been published. Kambin and colleagues [13, 16] reported an 88% excellent or good outcome rate with arthroscopic microdiscectomy, and others have reported similar success. In a prospective randomized study evaluating the efficacy of microscopic disc surgery compared with endoscopic disc extraction, Mayer and Brock [17] achieved favorable outcomes with minimal complications using the percutaneous arthroscopic technique.

### 2.4. Laparoscopic Lumbar Spine Surgery

The modern era of laparoscopy began in the 1980s when Kurt Semm performed the first appendectomy in Germany [18]. Semm, a physician and an engineer, developed many tools that are still in use. The first human laparoscopic cholecystectomy was performed in 1987 by Dubois et al. [19]. With the advantages of laparoscopic exposure being championed by urological, gynecological, and general surgeons, it is natural that spine surgeons would consider extending these technologies to the anterior lumbar spine. The advantages of transperitoneal laparoscopic spinal surgery include improved observation of surgical anatomic features, marked reductions in postoperative pain, early hospital discharges, and reduced incidence of postoperative ileus. In 1991, Obenchain [20] reported the first use of a laparoscopic approach to the lumbar spine for a discectomy. Regan et al. [21] described the technique and reported preliminary results for laparoscopic anterior lumbar fusion. Gaur [22] was the first to describe an endoscopic retroperitoneal approach for urological procedures, and Fessler first described this retroperitoneal endoscopic approach in the lumbar spine in 1992 and a lumbar fusion via this technique in 1997 [23]. McAfee et al. demonstrated that they too had good results in eighteen patients in 1998 [24].

## 3. Minimally Invasive Methods Treating Disc Pathology

### 3.1. Chemonucleolysis

In 1941, Eugene Jansen and Arnold Balls isolated chymopapain from crude papain which had been derived from the latex of* Carica papaya* [25]. Lewis Thomas intravenously injected rabbits with crude papain in 1956 and observed that their ears drooped [26]. Intrigued by its potential uses, Smith et al., in 1963, were the first to inject chymopapain in a herniated nucleus pulposus for the treatment of sciatica [27]. This process, called chemonucleolysis, alters the characteristics of the nucleus pulposus by liberation of chondroitin sulfate and keratin sulfate through hydrolysis of noncollagenous mucopolysaccharide proteins, leading to polymerization of the nucleus pulposus.

Three double-blinded studies reported the efficacy of chemonucleolysis to be 74% and in 13 retrospective studies it was reported as 77% [28–30]. Nordby et al. had an 87.2% success rate in over 3000 patients, but as Phase III trials demonstrated mixed results, chemonucleolysis was not uniformly adapted in orthopedic and neurosurgical communities [30–32]. However, literature reviews demonstrate that chemonucleolysis can still be safely and effectively used for treatment of disc herniation as long patients are carefully selected and a proper injection technique is used [30, 33–36].

### 3.2. Percutaneous Laser Discectomy

Ascher and Heppner [37] were the first to use the technique of percutaneous laser discectomy to treat lumbar disc disease. With fluoroscopic verification of the level and placement of the needle and coupling through a fiber, laser energy is passed into the disc space. The laser energy is transmitted in short bursts to avoid excessive heating of the adjacent tissues. Their technique involved measuring the intradiscal pressure before and after laser discectomy using a saline manometer. They postulated that the removal of even a small volume of tissue from the disk caused a corresponding decrease in intradiscal pressure [38].

The results of percutaneous laser disc decompression in cases involving back and leg pain with disc protrusions are still unclear. No controlled prospective studies have been performed to evaluate the results of percutaneous laser discectomy. The largest experience in the literature was reported by Choy et al. [39]. They reported an 87.4% excellent result rate in a study of 333 patients, with a mean follow-up of 26 months. Early experience with the KTP/532 laser device was reported by Davis [40], who achieved an 85% success rate. Yeung [41] reported good to excellent results in 86.4% with the KTP/532 device. Fiume et al. found no differences between the treated and control groups by analyzing responses to pain questionnaires or by becoming aware of physical symptoms [42].

### 3.3. Intradiscal Electrical and Radiofrequency Thermocoagulation

Intradiscal electrical thermocoagulation (IDET) and percutaneous intradiscal radiofrequency thermocoagulation (PIRFT) have been used to treat primary discogenic back pain, mostly pain derived from internal disk disruption and annular tears. IDET involves threading a flexible heating electrode percutaneously into the disc, such that the electrode passes circumferentially around the inner surface of the disc. The heating of the electrode denatures the collagen of the annulus and coagulates the pain fibers supplying the annulus. PIRFT is thought to work via the same mechanism, except the heat is generated by energy from a radiofrequency probe.

In 2000, J. A. Saal and J. S. Saal [43] reported on 62 patients with low back pain treated with IDET; 71% of patients experienced a mean improvement of 3 points in their VAS back pain score. At two-year follow-up, Bogduk and Karasek found that patients with IDET did significantly better than those who did not receive the treatment with 54% of treated patients achieving at least 50% relief of their pain and no longer using opioids and returning to work [44]. Two prospective randomized trials demonstrated pain relief of IDET, although these studies were limited by total sample sizes of 64 and 57 patients [45, 46]. In 2005, Kapural et al. prospectively matched 42 patients for either IDET or PIRFT and concluded that patients who received IDET had significantly improved pain scores than those who received PIRFT [47]. Recently the treatments of IDET and PIFRT have fallen out of favor as recent systematic reviews concluded that there was a paucity of evidence demonstrating benefit [48, 49].

## 4. Bone Augmentation

The spine is composed of a rich trabecular lattice of cancellous bone encased in a hard cortical shell. Moreover, the spine is exposed to degrees of compressive loads and tensile stresses that are in symbiotic biomechanical play with the inner and outer matrices of the vertebral bodies. Osteoporotic bone loss or neoplastic invasion of the vertebral bodies results in erosion of the cancellous network and development of vertebral compression fractures (VCFs), which can contribute to debilitating pain, neurological deficit, gross spinal instability, and resultant deformity. Surgical management involves considerable risk because of the high prevalence of significant comorbidities in these patients. Surgical decompression and reconstruction involves internal fixation using screws, plates, wires, cages, or rods and requires extensive surgical exposure. The time required for recuperation from open fixation procedures can be lengthy. Obtaining satisfactory fixation in osteoporotic bone can be technically difficult, and the failure rate for spinal arthrodesis is significant.

### 4.1. Vertebroplasty

In an attempt to reduce such invasive operative treatment, percutaneous vertebroplasty (PVP) was developed in 1984 by Galibert and Déramond [50] in France as a minimally invasive outpatient procedure to offer immediate pain relief by the injection of polymethylmethacrylate (PMMA) bone cement into the vertebral body through a transpedicular approach. Although rapidly popularized in Europe, PVP was not adopted in the United States until 1994 [51].

### 4.2. Kyphoplasty

In an effort to reduce the high incidence of cement extravasation and detrimental sequelae such as infection, cement toxicity, and adjacent fracture development due to altered sagittal balance, kyphoplasty was developed in the mid-1990s by Garfin et al. [52]. Kyphoplasty implements inflatable bone tamps inserted via a bilateral percutaneous transpedicular approach. Balloon inflation ultimately both decreases intravertebral pressure by creating a cavity which is filled with PMMA and also distracts the vertebral endplates to restore vertebral height [53].

### 4.3. Application of Image-Guidance Systems in the Spine

Image-guidance systems are widely used in intracranial surgery and have been adapted to assist with screw placement since the mid-1990s. The use of image-guidance systems for pedicle screw placement is intended to improve overall accuracy. These systems typically rely upon precise localization of the bony anatomy with preoperative computed tomography (CT). In this way, the transverse width, longitudinal depth, and trajectory angle can be easily measured on a computer-assisted work station.

Nolte et al. [54] described the principles of computer-assisted pedicle screw fixation. The overall accuracy of their system was 1.74 mm, using CT scans with 22 mm slice increments. Intraoperative surgical exposure of the posterior vertebral elements was performed using standard surgical techniques. An infrared camera (Optotrak, Northern Digital, Waterloo, Ontario, Canada) tracked specific instruments (i.e., pedicle probe, awl, and space pointer) equipped with light-emitting diodes. The dynamic reference was fixed to the spinous process of the vertebra to be instrumented. Normal bony landmarks and their correlations with the images confirmed the calibration accuracy. Using that computerized system, they reported a pedicle screw misplacement rate of 4.3% under clinical conditions.

Choi et al. [55] reported the use of computer-assisted fluoroscopic targeting for pedicle screw fixation. They described a system in which the pedicle entry site and the depth of insertion were determined by intraoperative anteroposterior and lateral fluoroscopic scans. Those authors compared the accuracy of placement with the fluoroscopy-guided system versus the CT-guided system and observed no significant differences.

## 5. Expanding Minimally Invasive Indications

**Figure 1:**
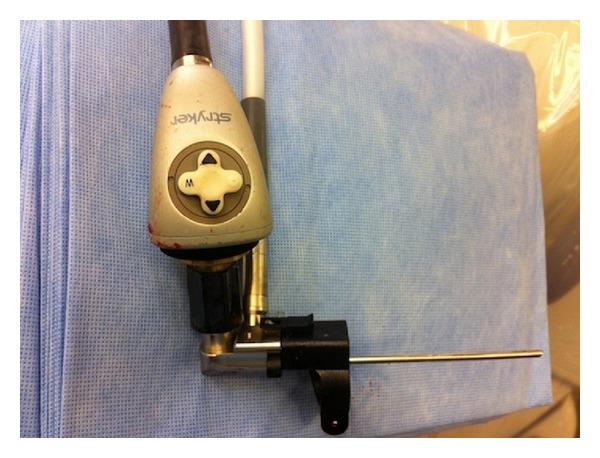
A view of the microendoscope.

In the last few years, the indications for minimally invasive spine surgery have increased profoundly as surgeons' proficiency with techniques has improved (Figure 1). Microendoscopic and microscopic foraminotomies, discectomies, and laminectomies via a lateral incision and tubular dilation-retraction have replaced the standard open foraminotomies, discectomies, and laminectomies [56–59]. Surgeons' increasing comfort with the tube allows them to perform these procedures in the cervical, thoracic, and lumbar spine.

**Figure 2:**
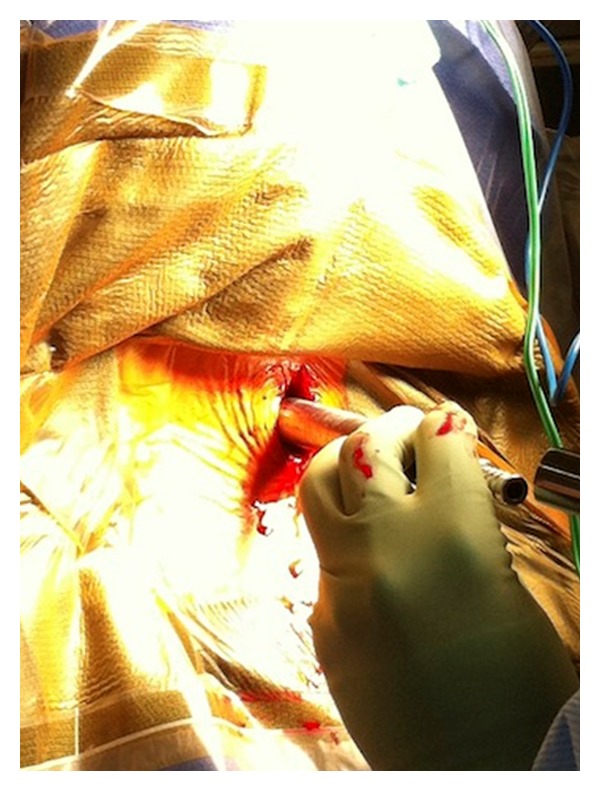
Tubular dilation in the microendoscopic foraminotomy.

**Figure 3:**
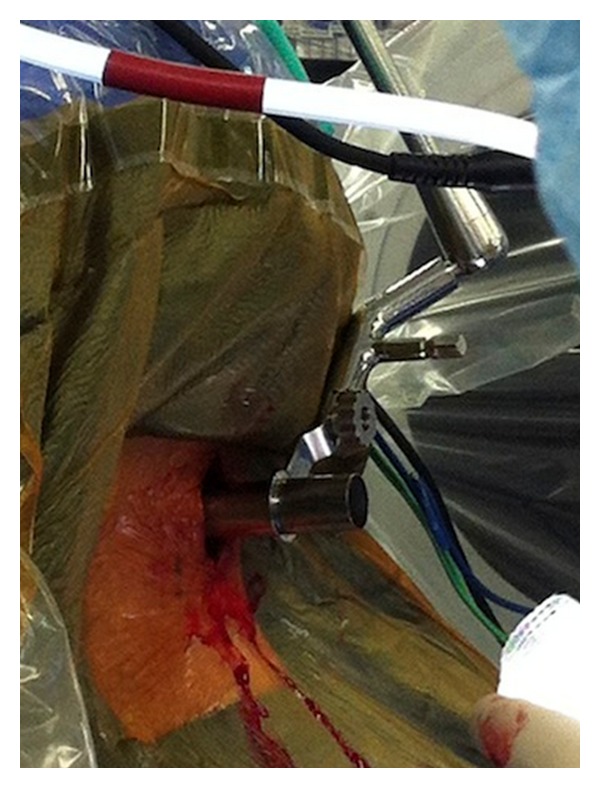
Docking of final tubular retractor in microendoscopic foraminotomy.

**Figure 4:**
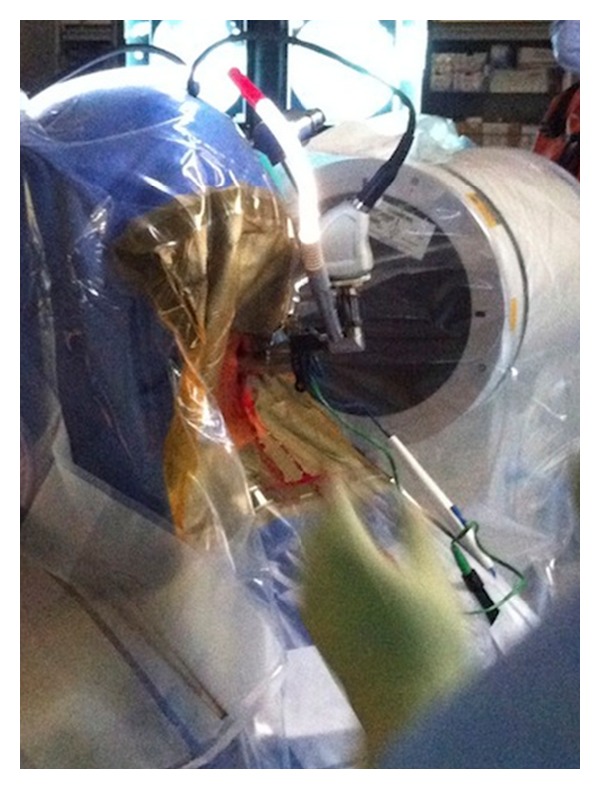
Docking of microendoscope to final tubular retractor with light source.

Currently, most of these minimally invasive procedures involve using progressive dilators to dilate through the muscle onto the facet at the desired level (Figure 2). The interlaminar space can be visualized through the largest dilation tube and the inferior edge of the lamina is removed using a kerrison or drill (Figure 3). Often a portion of the medial facet will be removed for foraminotomies and discectomies [58, 60]. Suction retractors allow gentle movement of the nerve roots for access to the disc for discectomies. Long and angled instruments have been developed to allow visualization and dissection, including drills, knives, pituitaries, and kerrisons. For visualization, the endoscope or microscope can be used (Figure 4).

In the lumbar spine, angulation of the endoscope or microscope medially allows decompression of the contralateral lateral recess, and minimally invasive laminectomies can be achieved [61]. If greater visualization is necessary, a portion of the inferior spinous process can be removed. Patients have been shown to have similar outcomes in these procedures if not better than those of the traditional open techniques [62–66].

For thoracic disc resection, thoracoscopy nor thoracotomy is necessarily required, especially for soft discs [67, 68]. Thoracic disc removal by microendoscopy involves an incision 3-4 m lateral to midline in combination with partial facetectomy and medical angulation of the endoscope or microscope allows visualization of the disc without retraction of the thoracic cord [69, 70]. Minimally invasive retropleural approaches using tubular retractor systems for central or calcified thoracic disc herniation have been described via a lateral mini-open approach [71].

When fusion with an interbody in these cases is required, the entire inferior facet will be removed via osteotomes or drilling to allow placement of an interbody, all through a larger tube [72]. A skin and fascial entry around 3 cm from the spinous process in minimally invasive transforaminal lumbar fusions, instead of around 1.5 cm from the spinous process in minimally invasive discectomies and laminectomies, allows enough of a lateral entry to medially angle an interbody across the disc space [73, 74]. The same incision site is used as an entry to place pedicle screws with image guidance or percutaneously using a combination of AP and lateral fluoroscopy. If bilateral fixation is desired, dilation is performed via a separate incision on the other side of the spinous processes [75, 76]. Minimally invasive transforaminal lateral interbody fusions can prevent a large amount of muscle dissection, exposure to microbes, and creation of dead space that often occurs from exposing the transverse processes in traditional transforaminal lateral interbody fusion techniques, with similar or better outcomes [77–81].

Minimally invasive techniques have been used to treat multiple pathologies from synovial cysts to metastatic tumors to epidural abscesses [82–85]. For traumatic fractures as well as pathological fractures, minimally invasive corpectomies with reconstruction of the anterior column are possible [86–88]. Although new case reports appear in spine journals regularly, here we focus on two areas of minimally invasive spine surgery that have expanded rapidly in the last ten years, spinal deformity and intradural pathology.

### 5.1. Spinal Deformity

In 2006, Ozgur et al. described an extreme lateral interbody fusion technique (XLIF; NuVasive, Inc.), a mini-open version of the retroperitoneal endoscopic technique, that had been previously presented by Pimenta [89]. Through the lateral retroperitoneal fat and the psoas muscle, access to the lateral lumbar spine could be obtained to treat degenerative disc disease and provided an anterior lumbar interbody fusion. No access surgeon was needed and a large interbody graft could be placed without bone drilling. The same extreme lateral approach has also been used for resection of far lateral discs [90]. The ability to place multiple interbody grafts through this lateral technique has allowed surgeons to proficiently correct coronal deformities in a minimally invasive way [91–94]. The transpsoas approach has become a mainstay of minimally invasive spine surgeons.

**Figure 5:**
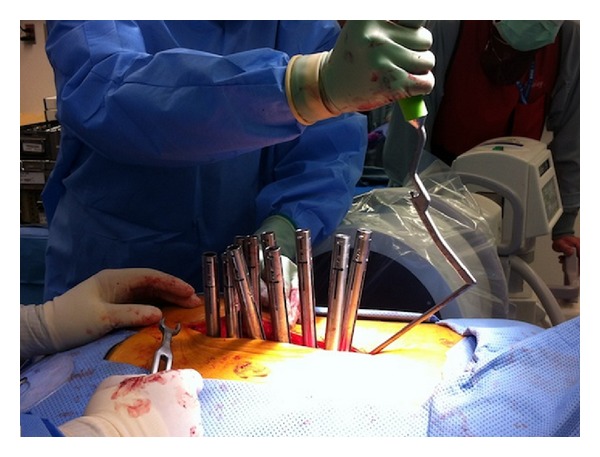
Multilevel lumbar fusion via hybrid technique.

The potential of minimally invasive deformity correction has burgeoned a milieu of new techniques and retractor systems. Minimally invasive deformity correction usually includes a transpsoas approach at multiple levels of discectomy, anterior release and interbody fusion, followed by a separate procedure, often on another day, of posterior multilevel percutaneous pedicle screw and rod placement [95–99]. Occasionally, the presacral approach for fixation and interbody fusion at L5-S1 and L4-L5 is used [100–102]. Placement of minimally invasive percutaneous iliac screws also allows for longer-segment deformity fusions with significant biomechanical strength advantages [103]. Preoperative planning and identifying the suitable indications for minimally invasive deformity surgery are imperative as certain deformities such as patients with large Cobb angles and high-grade spondylolisthesis can be difficult to correct with less complication than open techniques. For example, although minimally invasive osteotomies are possible, many surgeons still prefer to perform open traditional techniques to perform them [100, 104]. Appropriate correction of sagittal balance and improvements in lordosis can be difficult to achieve with percutaneous screws alone, and thus some surgeons are now using a combination of mini-open and minimally invasive techniques (Figure 5) [105, 106].

### 5.2. Intradural Pathology

Minimally invasive techniques have expanded in the role of spinal tumor resection. Surgeons may now use the endoscope or the microscope for visualization in many of these procedures. In 1955, Malis used a binocular microscope intraoperatively in conjunction with bipolar coagulation to aid him with his surgical approach [107]. A unilateral approach to exposure and spinal tumor resection had been championed by Chiou et al. in 1989 and Yaşargil et al. in 1991 [108, 109]. Other authors had followed in performing unilateral limited laminectomies [110, 111]. In 2004, Pompili et al. emphasized this limited approach in thoracolumbar neurofibroma resection [112]. In 2006, Tredway et al. published the first series of minimally invasive resection of intradural tumors using a unilateral dilation technique and self-retaining retractor system that included 6 patients [113]. Other spine surgeons have since adopted this technique and made their own improvements, including those for dural closure [114–116].

As surgeons' comfort with intradural pathology has improved so have their minimally invasive techniques to treat intradural pathologies other than tumors. Other intradural pathologies such as dural arteriovenous fistulas have been treated using minimally invasive techniques as well. Day described treatment of a dural arteriovenous fistula via a partial open hemilaminectomy in 2008 [117]. In 2012, Desai et al. described one patient and Patel et al. described seven patients for whom intradural arteriovenous fistulas treated through a tubular retractor system had good results [118, 119]. Obliteration of the fistula can be confirmed with digital subtraction angiography or with indocyanine green angiography. This work has been continued at other centers [120].

## 6. Conclusion

Although those techniques described and many more minimally invasive techniques are possible, they have only been slowly adopted by spine surgeons as many do not have enough exposure in learning the techniques. The rapid development of this technology requires active and continual learning on the part of spine surgeons to stay up to date. Many spine surgeons do not have the experience to deal with the difficulties that may arise in minimally invasive cases and thus will convert to open surgeries too early to avoid complications. As minimally invasive techniques have progressed extensively over the last 50 years, it is imperative that training programs instill these techniques early on, as they can provide improved outcomes for surgeons and their patients.

## References

[1] Burman MS (1931). Myeloscopy or the direct visualization of the spinal cord and its contents. *The Journal of Bone & Joint Surgery*.

[2] Pool JL (1938). Direct visualization of dorsal nerve roots of the cauda equina by means of a myeloscope. *Archives of Neurology and Psychiatry*.

[3] Pool JL (1942). Myeloscopy: intraspinal endoscopy. *Surgery*.

[4] Ooi Y, Satoh Y, Morisaki N (1973). Myeloscopy, possibility of observing lumbar intrathecal space by use of an endoscope. *Endoscopy*.

[5] Ooi Y, Satoh Y, Mikanagi K, Morisaki N (1977). Myeloscopy. *No To Shinkei*.

[6] Jacobeus HC (1910). Possibility of the use of the cystoscope for investigation of serous cavities. *Munchener Medizinische Wochenschrift*.

[7] Jacobeus HC (1921). The practical importance of thoracoscopy in surgery of the chest. *Surgery, Gynecology & Obstetrics*.

[8] Mack MJ, Regan JJ, Bobechko WP, Acuff TE (1993). Application of thoracoscopy for diseases of the spine. *Annals of Thoracic Surgery*.

[9] Rosenthal D, Rosenthal R, de Simone A (1994). Removal of a protruded thoracic disc using microsurgical endoscopy: a new technique. *Spine*.

[10] Ottolenghi CE (1955). Diagnosis of orthopaedic lesions by aspiration biopsy; results of 1,061 punctures. *The Journal of Bone and Joint Surgery. American*.

[11] Craig FS (1956). Vertebral-body biopsy. *The Journal of Bone and Joint Surgery. American*.

[12] Hijikata S, Yamgishi M, Nakayama T, Oomon K (1975). Percutaneous discectomy: a new treatment method for lumbar disc herniation. *Journal of Toden Hospital*.

[13] Kambin P, Gellman H (1983). Percutaneous lateral discectomy of the lumbar spine. A preliminary report. *Clinical Orthopaedics and Related Research*.

[14] Onik G, Helms CA, Ginsburg L (1985). Percutaneous lumbar diskectomy using a new aspiration probe. *American Journal of Roentgenology*.

[15] Kambin P (1992). Arthroscopic microdiscectomy. *Arthroscopy*.

[16] Kambin P, Sampson S (1986). Posterolateral percutaneous suction-excision of herniated lumbar intervertebral discs. Report of interim results. *Clinical Orthopaedics and Related Research*.

[17] Mayer HM, Brock M (1993). Percutaneous endoscopic discectomy: surgical technique and preliminary results compared to microsurgical discectomy. *Journal of Neurosurgery*.

[18] Semm K (1983). Endoscopic appendectomy. *Endoscopy*.

[19] Dubois F, Icard P, Berthelot G, Levard H (1990). Coelioscopic cholecystectomy. Preliminary report of 36 cases. *Annals of Surgery*.

[20] Obenchain TG (1991). Laparoscopic lumbar discectomy: case report. *Journal of Laparoendoscopic Surgery*.

[21] Regan JJ, McAfee PC, Guyer RD, Aronoff RJ (1996). Laparoscopic fusion of the lumbar spine in a multicenter series of the first 34 consecutive patients. *Surgical Laparoscopy & Endoscopy*.

[22] Gaur DD (1992). Laparoscopic operative retroperitoneoscopy: use of a new device. *Journal of Urology*.

[23] Fessler RG Endoscopically assisted retroperitoneal lumbar fusion.

[24] McAfee PC, Regan JJ, Peter Geis W, Fedder IL (1998). Minimally invasive anterior retroperitoneal approach to the lumbar spine. Emphasis on the lateral BAK. *Spine*.

[25] Jansen EF, Balls AK (1941). Chymopapain: a new crystalline proteinase from papaya latex. *The Journal of Biological Chemistry*.

[26] Thomas L (1956). Reversible collapse of rabbit ears after intravenous papain, and prevention of recovery by cortisone. *The Journal of Experimental Medicine*.

[27] Smith L, Garvin PJ, Gesler RM, Jennings RB (1963). Enzyme dissolution of the nucleus pulposus. *Nature*.

[28] Fraser RD (1982). Chymopapain for the treatment of intervertebral disc herniation. A preliminary report of a double-blind study. *Spine*.

[29] Dabezies EJ, Langford K, Morris J, Shields CB, Wilkinson HA (1988). Safety and efficacy of chymopapain (Discase) in the treatment of sciatica due to a herniated nucleus pulposus. Results of a randomized, double-blind study. *Spine*.

[30] Javid MJ, Nordby EJ, Ford LT (1983). Safety and efficacy of chymopapain (chymodiactin) in herniated nucleus pulposus with sciatica. Results of a randomized, double-blind study. *Journal of the American Medical Association*.

[31] Nordby EJ, Javid MJ (2000). Continuing experience with chemonucleolysis. *Mount Sinai Journal of Medicine*.

[32] Nordby EJ, Brown MD (1977). Present status of chymopapain and chemonucleolysis. *Clinical Orthopaedics and Related Research*.

[33] Javid MJ (1996). Postchemonucleolysis discectomy versus repeat discectomy: a prospective 1- to 13-year comparison. *Journal of Neurosurgery*.

[34] Fraser RD (1984). Chymopapain for the treatment of intervertebral disc herniation. The final report of a double-blind study. *Spine*.

[35] Gunzburg R, Fraser RD, Moore R, Vernon-Roberts B (1993). An experimental study comparing percutaneous discectomy with chemonucleolysis. *Spine*.

[36] Gogan WJ, Fraser RD (1992). Chymopapain: a 10-year, double-blind study. *Spine*.

[37] Ascher PW, Heppner F (1984). CO_2_-laser in neurosurgery. *Neurosurgical Review*.

[38] Ascher PW (1985). Status quo and new horizons of laser therapy in neurosurgery. *Lasers in Surgery and Medicine*.

[39] Choy DSJ, Ascher PW, Saddekni S (1992). Percutaneous laser disc decompression: a new therapeutic modality. *Spine*.

[40] Davis JK (1992). Early experience with laser disc decompression. A percutaneous method. *Journal of the Florida Medical Association*.

[41] Yeung AT (2000). The evolution of percutaneous spinal endoscopy and discectomy: state of the art. *Mount Sinai Journal of Medicine*.

[42] Fiume D, Parziale G, Rinaldi A, Sherkat S (1994). Automated percutaneous discectomy in herniated lumbar discs treatment: experience after the first 200 cases. *Journal of Neurosurgical Sciences*.

[43] Saal JA, Saal JS (2000). Intradiscal electrothermal treatment for chronic discogenic low back pain: a prospective outcome study with minimum 1-year follow-up. *Spine*.

[44] Bogduk N, Karasek M (2002). Two-year follow-up of a controlled trial of intradiscal electrothermal anuloplasty for chronic low back pain resulting from internal disc disruption. *Spine Journal*.

[45] Pauza KJ, Howell S, Dreyfuss P, Peloza JH, Dawson K, Bogduk N (2004). A randomized, placebo-controlled trial of intradiscal electrothermal therapy for the treatment of discogenic low back pain. *Spine Journal*.

[46] Freeman BJ, Fraser RD, Cain CM, Hall DJ, Chapple DC (2005). A randomized, double-blind, controlled trial: intradiscal electrothermal therapy versus placebo for the treatment of chronic discogenic low back pain. *Spine*.

[47] Kapural L, Hayek S, Malak O, Arrigain S, Mekhail N (2005). Intradiscal thermal annuloplasty versus intradiscal radiofrequency ablation for the treatment of discogenic pain: a prospective matched control trial. *Pain Medicine*.

[48] Helm S, Hayek SM, Benyamin R, Manchikanti L (2009). Systematic review of the effectiveness of thermal annular procedures in treating discogenic low back pain. *Pain Physician*.

[49] Urrútia G, Kovacs F, Nishishinya MB, Olabe J (2007). Percutaneous thermocoagulation intradiscal techniques for discogenic low back pain. *Spine*.

[50] Galibert P, Déramond H (1990). Percutaneous acrylic vertebroplasty as a treatment of vertebral angioma as well as painful and debilitating diseases. *Chirurgie*.

[51] Barr JD, Barr MS, Lemley TJ, McCann RM (2000). Percutaneous vertebroplasty for pain relief and spinal stabilization. *Spine*.

[52] Garfin SR, Yuan HA, Reiley MA (2001). New technologies in spine: kyphoplasty and vertebroplasty for the treatment of painful osteoporotic compression fractures. *Spine*.

[53] Dudeney S, Lieberman IH, Reinhardt M-K, Hussein M (2002). Kyphoplasty in the treatment of osteolytic vertebral compression fractures as a result of multiple myeloma. *Journal of Clinical Oncology*.

[54] Nolte L-P, Zamorano LJ, Jiang Z, Wang Q, Langlotz F, Berlemann U (1995). Image-guided insertion of transpedicular screws: a laboratory set-up. *Spine*.

[55] Choi WW, Green BA, Levi ADO (2000). Computer-assisted fluoroscopic targeting system for pedicle screw insertion. *Neurosurgery*.

[56] Fessler RG, Khoo LT (2002). Minimally invasive cervical microendoscopic foraminotomy: an initial clinical experience. *Neurosurgery*.

[57] Celestre PC, Pazmiño PR, Mikhael MM (2012). Minimally invasive approaches to the cervical spine. *Orthopedic Clinics of North America*.

[58] Christie SD, Song JK (2006). Minimally invasive lumbar discectomy and foraminotomy. *Neurosurgery Clinics of North America*.

[59] Thongtrangan I, Le H, Park J, Kim DH (2004). Minimally invasive spinal surgery: a historical perspective. *Neurosurgical Focus*.

[60] O’Toole JE, Eichholz KM, Fessler RG (2007). Minimally invasive far lateral microendoscopic discectomy for extraforaminal disc herniation at the lumbosacral junction: cadaveric dissection and technical case report. *Spine Journal*.

[61] An HS, Andersson G, Lieberman I, Riew D, Transfeldt E (2000). Minimally invasive surgery for lumbar degenerative disorders—part II: degenerative disc disease and lumbar stenosis. *American Journal of Orthopedics*.

[62] Lee P, Liu JC, Fessler RG (2011). Perioperative results following open and minimally invasive single-level lumbar discectomy. *Journal of Clinical Neuroscience*.

[63] Dasenbrock HH, Juraschek SP, Schultz LR (2012). The efficacy of minimally invasive discectomy compared with open discectomy: a meta-analysis of prospective randomized controlled trials. *Journal of Neurosurgery. Spine*.

[64] Rahman M, Summers LE, Richter B, Mimran RI, Jacob RP (2008). Comparison of techniques for decompressive lumbar laminectomy: the minimally invasive versus the “classic” open approach. *Minimally Invasive Neurosurgery*.

[65] Karnezis IA (2008). Minimally invasive therapeutic interventional procedures in the spine: an evidence-based review. *Surgical Technology International*.

[66] Allen RT, Garfin SR (2010). The economics of minimally invasive spine surgery: the value perspective. *Spine*.

[67] Perez-Cruet MJ, Kim B-S, Sandhu F, Samartzis D, Fessler RG (2004). Thoracic microendoscopic discectomy. *Journal of Neurosurgery. Spine*.

[68] Falavigna A, Piccoli Conzatti L (2013). Minimally invasive approaches for thoracic decompression from discectomy to corpectomy. *Journal of Neurosurgical Sciences*.

[69] Isaacs RE, Podichetty VK, Sandhu FA (2005). Thoracic microendoscopic discectomy: a human cadaver study. *Spine*.

[70] Eichholz KM, O’Toole JE, Fessler RG (2006). Thoracic microendoscopic discectomy. *Neurosurgery Clinics of North America*.

[71] Kasliwal M, Deutsch H (2011). Minimally invasive retropleural approach for central thoracic disc herniation. *Minimally Invasive Neurosurgery*.

[72] Park P, Foley KT (2008). Minimally invasive transforaminal lumbar interbody fusion with reduction of spondylolisthesis: technique and outcomes after a minimum of 2 years’ follow-up. *Neurosurgical Focus*.

[73] Wang J, Zhou Y, Zhang ZF, Li CQ, Zheng WJ, Liu J (2010). Comparison of one-level minimally invasive and open transforaminal lumbar interbody fusion in degenerative and isthmic spondylolisthesis grades 1 and 2. *European Spine Journal*.

[74] Karikari IO, Isaacs RE (2010). Minimally invasive transforaminal lumbar interbody fusion: a review of techniques and outcomes. *Spine*.

[75] Chen KS, Than KD, Lamarca F, Park P (2013). Minimally invasive unilateral approach for bilateral decompression of spinal stenosis and modified transforaminal lumbar interbody fusion for degenerative spondylolisthesis. *Neurosurgical Focus*.

[76] Dahdaleh NS, Nixon AT, Lawton CD, Wong AP, Smith ZA, Fessler RG (2013). Outcome following unilateral versus bilateral instrumentation in patients undergoing minimally invasive transforaminal lumbar interbody fusion: a single-center randomized prospective study. *Neurosurgical Focus*.

[77] Lawton CD, Smith ZA, Nixon AT (2014). The effect of surgical level on self-reported clinical outcomes after minimally invasive transforaminal lumbar interbody fusion: L4-L5 versus L5-S1. *World Neurosurgery*.

[78] Seng C, Siddiqui MA, Wong KP (2013). Five-year outcomes of minimally invasive versus open transforaminal lumbar interbody fusion: a matched-pair comparison study. *Spine*.

[79] Parker SL, Adogwa O, Bydon A, Cheng J, McGirt MJ (2012). Cost-effectiveness of minimally invasive versus open transforaminal lumbar interbody fusion for degenerative spondylolisthesis associated low-back and leg pain over two years. *World Neurosurgery*.

[80] Parker SL, Mendenhall SK, Shau DN (2013). Minimally invasive versus open transforaminal lumbar interbody fusion (tlif) for degenerative spondylolisthesis: comparative effectiveness and cost-utility analysis. *World Neurosurgery*.

[81] Mcgirt MJ, Parker SL, Lerner J, Engelhart L, Knight T, Wang MY (2011). Comparative analysis of perioperative surgical site infection after minimally invasive versus open posterior/transforaminal lumbar interbody fusion: analysis of hospital billing and discharge data from 5170 patients. *Journal of Neurosurgery. Spine*.

[82] Sandhu FA, Santiago P, Fessler RG (2004). Minimally invasive surgical treatment of lumbar synovial cysts. *Neurosurgery*.

[83] Safavi-Abbasi S, Maurer AJ, Rabb CH (2013). Minimally invasive treatment of multilevel spinal epidural abscess: technical note. *Journal of Neurosurgery. Spine*.

[84] Schultz KD, Comey CH, Haid RW (2001). Pyogenic spinal epidural abscess: a minimally invasive technique for multisegmental decompression. *Journal of Spinal Disorders*.

[85] Roselli R, Iacoangeli M, Pompucci A (1998). Anterior cervical epidural abscess treated by endoscopy-assisted minimally invasive microsurgery via posterior approach. *Minimally Invasive Neurosurgery*.

[86] Smith ZA, Wong AP, El Ahmadieh TY (2012). Minimally invasive thoracic corpectomy: 3-dimensional operative video of a direct lateral approach to decompression and anterior column reconstruction. *Neurosurgery*.

[87] Lall RR, Smith ZA, Wong AP, Miller D, Fessler RG (2012). Minimally invasive thoracic corpectomy: surgical strategies for malignancy, trauma, and complex spinal pathologies. *Minimally Invasive Surgery*.

[88] Eck JC (2011). Minimally invasive corpectomy and posterior stabilization for lumbar burst fracture. *Spine Journal*.

[89] Ozgur BM, Aryan HE, Pimenta L, Taylor WR (2006). Extreme Lateral Interbody Fusion (XLIF): a novel surgical technique for anterior lumbar interbody fusion. *Spine Journal*.

[90] Madhok R, Kanter AS (2010). Extreme-lateral, minimally invasive, transpsoas approach for the treatment of far-lateral lumbar disc herniation. *Journal of Neurosurgery. Spine*.

[91] Tormenti MJ, Maserati MB, Bonfield CM, Okonkwo DO, Kanter AS (2010). Complications and radiographic correction in adult scoliosis following combined transpsoas extreme lateral interbody fusion and posterior pedicle screw instrumentation. *Neurosurgical Focus*.

[92] Deukmedjian AR, Ahmadian A, Bach K, Zouzias A, Uribe JS (2013). Minimally invasive lateral approach for adult degenerative scoliosis: lessons learned. *Neurosurgical Focus*.

[93] Dakwar E, Cardona RF, Smith DA, Uribe JS (2010). Early outcomes and safety of the minimally invasive, lateral retroperitoneal transpsoas approach for adult degenerative scoliosis. *Neurosurgical Focus*.

[94] Amin BY, Mummaneni PV, Ibrahim T, Zouzias A, Uribe J (2013). Four-level minimally invasive lateral interbody fusion for treatment of degenerative scoliosis. *Neurosurgical Focus*.

[95] Anand N, Rosemann R, Khalsa B, Baron EM (2010). Mid-term to long-term clinical and functional outcomes of minimally invasive correction and fusion for adults with scoliosis. *Neurosurgical Focus*.

[96] Anand N, Baron EM, Thaiyananthan G, Khalsa K, Goldstein TB (2008). Minimally invasive multilevel percutaneous correction and fusion for adult lumbar degenerative scoliosis: a technique and feasibility study. *Journal of Spinal Disorders and Techniques*.

[97] Benglis DM, Elhammady MS, Levi AD, Vanni S (2008). Minimally invasive anterolateral approaches for the treatment of back pain and adult degenerative deformity. *Neurosurgery*.

[98] Wang MY, Mummaneni PV (2010). Minimally invasive surgery for thoracolumbar spinal deformity: initial clinical experience with clinical and radiographic outcomes. *Neurosurgical Focus*.

[99] Isaacs RE, Hyde J, Goodrich JA, Rodgers WB, Phillips FM (2010). A prospective, nonrandomized, multicenter evaluation of extreme lateral interbody fusion for the treatment of adult degenerative scoliosis: perioperative outcomes and complications. *Spine*.

[100] Anand N, Baron EM (2013). Minimally invasive approaches for the correction of adult spinal deformity. *European Spine Journal*.

[101] MacMillan M, Fessler RG, Gillespy M, Montgomery WJ (1996). Percutaneous lumbosacral fixation and fusion: anatomic study and two-year experience with a new method. *Neurosurgery Clinics of North America*.

[102] Gerszten PC, Tobler W, Raley TJ, Miller LE, Block JE, Nasca RJ (2012). Axial presacral lumbar interbody fusion and percutaneous posterior fixation for stabilization of lumbosacral isthmic spondylolisthesis. *Journal of Spinal Disorders and Techniques*.

[103] Wang MY, Williams S, Mummaneni PV, Sherman JD (2012). Minimally invasive percutaneous iliac screws: initial 24 case experience with CT confirmation. *Journal of Spinal Disorders and Techniques*.

[104] Voyadzis J-M, Gala VC, O’Toole JE, Eichholz KM, Fessler RG (2008). Minimally invasive posterior osteotomies. *Neurosurgery*.

[105] Wang MY (2013). Improvement of sagittal balance and lumbar lordosis following less invasive adult spinal deformity surgery with expandable cages and percutaneous instrumentation. *Journal of Neurosurgery. Spine*.

[106] Wang MY (2013). Less invasive mini-open adult spinal deformity surgery. *Neurosurgical Focus*.

[107] Epstein JA, Malis LI (1955). Compression of spinal cord and cauda equina in achondroplastic dwarfs. *Neurology*.

[108] Chiou SM, Eggert HR, Laborde G, Seeger W (1989). Microsurgical unilateral approaches for spinal tumour surgery: eight years’ experience in 256 primary operated patients. *Acta Neurochirurgica*.

[109] Yaşargil MG, Tranmer BI, Adamson TE, Roth P (1991). Unilateral partial hemi-laminectomy for the removal of extra- and intramedullary tumours and AVMs. *Advances and Technical Standards in Neurosurgery*.

[110] Sario-glu AC, Hanci M, Bozkus H, Kaynar MY, Kafadar A (1997). Unilateral hemilaminectomy for the removal of the spinal space-occupying lesions. *Minimally Invasive Neurosurgery*.

[111] Öktem IS, Akdemir H, Kurtsoy A, Koç RK, Menkü A, Tucer B (2000). Hemilaminectomy for the removal of the spinal lesions. *Spinal Cord*.

[112] Pompili A, Caroli F, Cattani F (2004). Unilateral limited laminectomy as the approach of choice for the removal of thoracolumbar neurofibromas. *Spine*.

[113] Tredway TL, Santiago P, Hrubes MR, Song JK, Christie SD, Fessler RG (2006). Minimally invasive resection of intradural-extramedullary spinal neoplasms. *Neurosurgery*.

[114] Ogden AT, Fessler RG (2009). Minimally invasive resection of intramedullary ependymoma: case report. *Neurosurgery*.

[115] Park P, Leveque J-C, Marca FL, Sullivan SE (2010). Dural closure using the U-clip in minimally invasive spinal tumor resection. *Journal of Spinal Disorders and Techniques*.

[116] Haji FA, Cenic A, Crevier L, Murty N, Reddy K (2011). Minimally invasive approach for the resection of spinal neoplasm. *Spine*.

[117] Diaz Day J (2008). Minimally invasive surgical closure of a spinal dural arteriovenous fistula. *Minimally Invasive Neurosurgery*.

[118] Patel NP, Birch BD, Lyons MK, DeMent SE, Elbert GA (2013). Minimally invasive intradural spinal dural arteriovenous fistula ligation. *World Neurosurgery*.

[119] Desai A, Bekelis K, Erkmen K (2012). Minimally invasive tubular retractor system for adequate exposure during surgical obliteration of spinal dural arteriovenous fistulas with the aid of indocyanine green intraoperative angiography: case report. *Journal of Neurosurgery. Spine*.

[120] Fontes RB, Tan LA, O'Toole JE (2013). Minimally invasive treatment of spinal dural arteriovenous fistula with the use of intraoperative indocyanine green angiography. *Neurosurgical Focus*.

